# Deficiency of Mouse FHR-1 Homolog, FHR-E, Accelerates Sepsis, and Acute Kidney Injury Through Enhancing the LPS-Induced Alternative Complement Pathway

**DOI:** 10.3389/fimmu.2020.01123

**Published:** 2020-06-19

**Authors:** Xiangru Li, Zhenhua Hao, Xiaorong Liu, Wei Li

**Affiliations:** ^1^Beijing Key Laboratory for Genetics of Birth Defects, Beijing Pediatric Research Institute, Beijing Children's Hospital, Capital Medical University, Beijing, China; ^2^MOE Key Laboratory of Major Diseases in Children, Beijing, China; ^3^Genetics and Birth Defects Control Center, National Center for Children's Health, Beijing, China; ^4^Department of Nephrology, Beijing Children's Hospital, Capital Medical University, National Center for Children's Health, Beijing, China

**Keywords:** sepsis, FHR-E, alternative complement pathway, acute kidney injury, LPS

## Abstract

Alternative complement pathway (AP) plays an important role in the development of sepsis, which is life threatening. Deficiency of factor H-related protein 1 (FHR-1), which is a regulator of AP, has been considered as a susceptible factor for atypical hemolytic uremic syndrome (aHUS) and other types of nephropathy when an inducer such as infection exists. However, the underlying mechanism of the disease development is largely unknown. There is no report on *CFHR1* gene knockout in any animal infection model and its function *in vivo* is still unclear. Here, a *Cfhr1* knockout mouse was generated for investigating AP in sepsis and sepsis-induced acute kidney injury (AKI). We found that murine FHR-1 homolog (FHR-E) deficiency enhanced lipopolysaccharide (LPS)-induced AP activation both *in vitro* and *in vivo* and that *Cfhr1* knockout mice exhibited more severe sepsis and AKI in response to LPS challenge. These results indicated that FHR-E deficiency promoted LPS-induced sepsis and AKI through AP over-activation, providing a mouse model for studying AP regulation and sepsis. This study revealed the function of FHR-E *in vivo*, which may further provide hints to the pathogenesis of FHR-1 deficiency-related diseases by enhancing LPS-induced AP activation.

## Introduction

Sepsis is a critical health condition with high mortality rate (1, 2) and acute kidney injury (AKI) is a common severe complication of sepsis (3). Gram-negative bacteria infection is a predominant cause of severe infection-triggered sepsis (4). Sepsis is an intricate process during which complement system is activated and proved to be double-edged with benefits and harms (5). The complement system is crucial in immune surveillance (6) and has extensive cross-talk with coagulation system and inflammation for homeostasis (7). It is triggered through three pathways among which the alternative complement pathway (AP) activation is responsible for more than 80% of terminal complement activation (8, 9). AP plays an important role in endotoxin clearance during the process of sepsis (10). Lack of AP activation in individuals predisposes to infection (11–13). However, it is also life-threatening when complement is excessively activated, and inhibition of its over-activation prevents organ injuries (14). Therefore, uncovering the precise regulatory mechanism of AP during sepsis may shed light on sepsis intervention. However, the mechanism of AP regulation during sepsis remains elusive.

Factor H (FH) and its related proteins (FHRs) are regulators of AP (15) and responsible for determining complement activating surfaces (16). FH, the major regulatory factor of AP (17), has been widely investigated, while functions of FHRs are unclear or controversial. FH and FHRs consist of different number of complement control protein modules (CCPs). All FHRs contain homologous CCPs to CCP19, 20 of FH which are responsible for ligand recognition (18). In general, FH functions as a cofactor of Factor I (FI) to cleave C3b and accelerate the decay of C3 convertase (19, 20), while FHRs act as the competitor of FH (16). However, different FHRs may have different functions. FHR-2 inhibits C3 convertase (21). FHR-3 displays the cofactor activity for FI (22). FHR-5 inhibits C3 convertase in fluid phase and displays cofactor activity for FI (23). Nevertheless, it remains elusive for the physiological functions of FHRs.

Mutations in FH and FHRs are associated with various diseases (24). *CFHR3-CFHR1* deletion increased the risk of atypical hemolytic uremic syndrome (aHUS) and systemic lupus erythematosus (SLE) (25–27). *CFHR3-CFHR1* deletion was proved to protect against IgA nephropathy (IgAN) (28) and age-related macular degeneration (AMD) (29). The precise mechanism of this contradictory effect is unclear. *In vitro* studies of factor H-related protein 1 (FHR-1) have shown that FHR-1 interferes with the regulation of FH by competing with FH and inhibits the activity of C5 convertase and the formation of terminal complement complex (TCC) (30). No obvious C3 and C5 regulatory activity at physiological concentration has been found in FHR-1 (16), albeit it is the most abundant FHR protein (18, 31). Healthy individuals with *CFHR3-CFHR1* deletion showed higher frequency of patrolling monocytes, plasmacytoid dendritic cells (DCs), and lower frequency of classical monocytes, myeloid DCs. Monocytes with *CFHR3-CFHR1* deletion secreted higher level of IL-1β in response to LPS challenge (32). Necrotic cells bound to FHR-1 promotes the secretion of TNF-α, IL-1β, IL-18, and IL-6 by monocytes (33). These reports suggest multiple effects of *CFHR1* deletion and highlight its complexity. The existence of relatively high frequency of healthy individuals with *CFHR3-CFHR1* deletion (34) indicates that other triggers are essential to amplify the effect of FHR-1 deficiency, such as infections (35, 36). However, there is no report on FHR-1 deficiency in any animal infection model.

**Figure 1:**
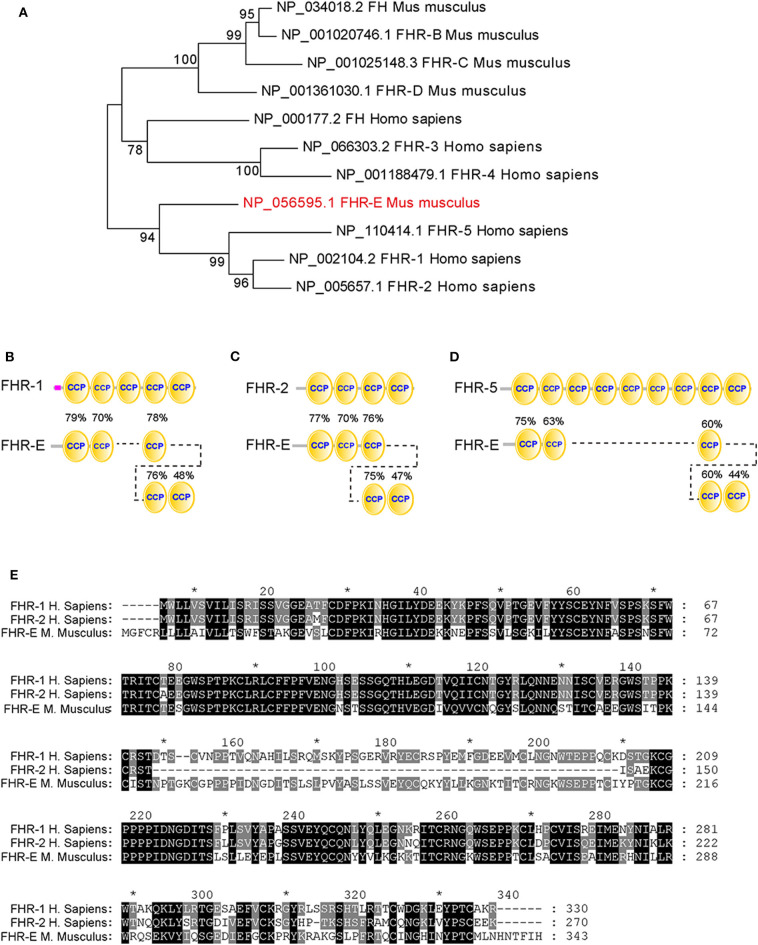
Homology analysis of different FHRs between human and mouse. **(A)** The evolutionary tree of different FHRs in human and mouse. The percentage of replicate trees in which the associated taxa clustered together in the bootstrap test (1,000 replicates) were shown next to the branches. **(B–D)** The similarity analyses between FHR-E and human FHR-1, FHR-2, and FHR-5. The percentages marked on these figures denote the similarities between the CCP domains of FHR-E and human FHR-1, FHR-2, and FHR-5. **(E)** The sequence alignment of FHR-E and human FHR-1 and FHR-2. * Indicates the number of amino acid. It was marked every twenty amino acids.

Three genes, *Fhrb, Fhrc*, and *Fhre (alias Cfhr1)*, and two pseudogenes, *Fhra and Fhrd*, were identified in mouse (37, 38). Recombinant FHR-A, FHR-B, and FHR-C were reported to interact with human C3d, suggesting that murine FHRs function as homologs of human FHRs (39). None of FHR-A, FHR-B, and FHR-C contain the critical dimerization domain that exists in human FHR-1, FHR-2, and FHR-5 (39), while FHR-E is predicted to contain it (37). Thus, FHR-E is more likely the murine homolog of human FHR-1 or FHR-2 (40). Nomenclature of human and mouse FHRs differs and there exist discrepancies in literature and existing database (41). Inferred from the homology analysis (Figure 1), we use mouse FHR-1 homolog FHR-E (NP_056595) for the protein encoded by *Cfhr1* (RefSeq# at NCBI: NM_015780, and a locus at MGI database: 2138169).

Homology analysis has shown the difference between human and murine FHRs (41, 42), suggesting that murine FHRs may function differently from their human counterparts. FHR-B, FHR-C, and FHR-E are functional FHRs in mouse. FHR-B and FHR-C were detected in mouse plasma with anti-FH antibody (38). Mouse FHR-B interacts with C3b and pentraxin 3 to enhance complement activation and necrotic cells promoted this activation (43). *Fhrc* mRNA was found completely absent in the liver of three lupus-prone mouse strains and one diabetic-prone mouse strain (44). FHR-C may function on specific surfaces (39). It is unknown whether FHR-E exists in murine plasma and how it functions in AP. Its human homolog, FHR-1, was reported to function as a competitor of FH (22, 30, 45) and its deletion was associated with various autoimmune diseases. However, its function *in vivo* and whether it has a regulatory role alone in the activation of AP remain unclear.

In this study, *Cfhr1* was deleted on C57BL/6 mouse to study the function of FHR-E on AP and the effect of FHR-E deficiency on LPS-induced sepsis. We found that FHR-E deficiency increased the mortality rate of LPS-induced sepsis and potentiated kidney injury through enhancing AP activation. Our data demonstrated a protective role of FHR-E during LPS-induced sepsis *in vivo* and highlighted its importance in AP regulation.

## Materials and Methods

### Mice

*Cfhr1* was deleted on C57BL/6 mouse by using the CRISPR/Cas9 system developed by Biocytogen Co. (Beijing, China). The heterozygous mouse was backcrossed with wild-type C57BL/6 for more than six generations. All the mice were bred in the animal facility of the Institute of Genetics and Developmental Biology (IGDB), Chinese Academy of Science. All procedures were approved by the Institutional Animal Care and Use Committee of IGDB. The age-matched wild-type (WT) or heterozygous littermates were used as controls. All experiments were conducted on the offspring of mice backcrossed more than six generations.

### Antibodies

The polyclonal antibody anti-FHR-E was generated in New Zealand white rabbit. The DNA fragment of the last three CCP domains (amino acids 146 to 343) of FHR-E was inserted into the expression vector pGEX-4T-1 and expressed in *E. coli* BL21 host cells. The recombinant protein was expressed as insoluble protein and separated by SDS-PAGE. The separated recombinant protein was used as an antigen to immunize the New Zealand white rabbit every other week. The rabbit was sacrificed 1 week after the fourth immunization and the sera was used as the anti-FHR-E polyclonal antibody. The rat anti-C3 (ab11862), rat anti-C5 (ab194637), sheep anti-FH (ab8842), and rabbit anti-fibrinogen (ab27913) were obtained from Abcam (Cambridge, UK). Purified rat anti-mouse C5a (558027) and biotin rat anti-mouse C5a (558028) were obtained from BD Pharmingen (California, USA).

### DNA Extraction and Genotyping

**Figure 2:**
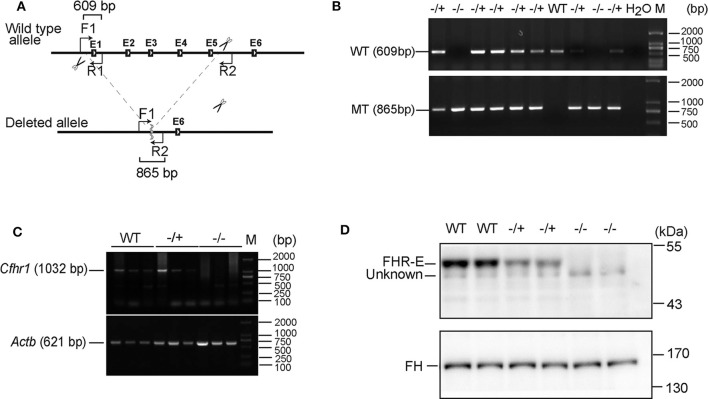
Verification of the absence of FHR-E in *Cfhr1*-KO mice. **(A)** The scheme of *Cfhr1* knockout strategy. F1, R1, and R2 indicate the primers used for genotyping. The expected bands amplified by two pairs of primers were 609 and 865 bp, respectively. **(B)** Verification of *Cfhr1* deletion from genome level. The upper bands were amplified with primers F1 and R1. These bands can only be amplified in wild-type and heterozygous mice and cannot be amplified in homozygous mice because of the absence of the sequence of primer R1. The lower bands were amplified with primers F1 and R2. These bands can only be amplified in heterozygous and homozygous mice. The sequence between primers F1 and R2 is too long to be amplified in wild-type mice. **(C)** Verification of *Cfhr1* deletion from RNA level. Hepatic RNA of mice of different genotypes was extracted and reversely transcribed. Primers spanning the open reading frame (ORF, 1,032 bp, NM_015780) of *Cfhr1* were used for RT-PCR. *Actb* was used as a semi-quantitative control. **(D)** Verification of FHR-E deficiency at the protein level. Plasma proteins of mice with different genotypes were separated by 8% SDS-PAGE gel. Proteins were transferred to the membrane and analyzed by Western blotting using anti-serum to mouse FHR-E, which was generated in rabbit immunized with a recombinant peptide of CCP 3–5 of FHR-E. An about 50 kDa specific FHR-E band was detected in WT and heterozygous mice, but not in homozygous mice. “Unknown” indicates an unknown protein recognized by anti-FHR-E antibody. Western blotting of FH was regarded as an internal control.

Mice were marked by cutting different toes and the toes were lysed by 250 μl of lysis buffer (Tris–HCl pH 8.0 100 mM, EDTA pH 8.0 5 mM, NaCl 200 mM, 0.2% SDS, and 0.1 mg/ml Proteinase K) at 55°C for 3 h. After transient centrifugation, 150 μl of 5 M NaCl was added and mixed thoroughly. The mixture was centrifuged at 12,000 rpm for 15 min and the supernatant was collected by adding double volume of ethanol and mixed thoroughly. The mixture was centrifuged at 12,000 rpm for 15 min and then the supernatant was discarded. The pellet was washed with 70% ethanol. After the liquid was evaporated, 100 μl of ddH_2_O was added to dissolve the precipitated DNA. One forward primer located on the upstream of the deleted sequence, one reverse primer located on the deleted region, and the other reverse primer located on the downstream of the deleted sequence were used for genotyping PCR assays. The primer's sequences are 5′-CAGTAAGACTGCAAGAGACATATG-3′, 5′-CTAAGAGCAACAGGCGACAG-3′, and 5′-CATTTTAAAAGAAAAATAAGCCAGCCA-3′, respectively. The amplified products are depicted in Figure 2A.

### Reverse Transcription-Polymerase Chain Reaction (RT-PCR)

Mouse hepatic RNA was extracted using RNeasy Mini Kit (Qiagen, Germantown, Germany) and reversely transcribed with Bio-Rad transcript cDNA synthesis kit (Bio-Rad, California, USA). The cDNA was used as templates to amplify *Cfhr1* and *Actb* (gene for β-actin). Primers of *Cfhr1* were 5′-ATGGGGTTCTGTCGCCTGTTGC-3′ and 5′-TCAATGAATAAACGTATTGTGA-3′. Primers of *Actb* were 5′-TGATGGTGGGAATGGGTCAGA-3′ and 5′-CCGCTCGTTGCCAATAGTGAT-3′.

### Immunoblotting

Equal volume of serum or plasma of mice was diluted 1:10 with PBS (Hyclone, Utah, USA). Equal volumes of diluted samples were separated with 8% SDS-PAGE gel and transferred onto polyvinylidene difluoride (PVDF) membrane (Millipore, Massachusetts, USA). The membrane was blocked with 5% defatted milk (BD, California, USA) for 1 h at room temperature and immunoblotted with specific antibody overnight at 4°C followed by incubation with HRP-conjugated secondary antibody (ZSGB-Bio, Beijing, China) for 1 h at room temperature. The membrane was visualized by ECL (Thermo Fisher Scientific, Massachusetts, USA or GE Healthcare, USA) and exposed with chemiluminescence apparatus (Beijing Sage Creation, Beijing, China). Protein bands' gray values were measured by NIH Image J.

### LPS Treatment *in vitro*

Serum was diluted by AP buffer (2.5 mM barbital, 1.5 mM sodium barbital, 144 mM NaCl, 7 mM MgCl_2_, and 10 mM EGTA, pH 7.2–7.4). Ten-fold diluted serum was incubated with 0.125 mg/ml LPS O111:B4 (Sigma-Aldrich, Missouri, USA) or equal volume of PBS as control at 37°C for 30 min. The reactions were stopped by 50 mM EDTA. The samples were analyzed with 8% SDS-PAGE under reducing condition and protein bands' gray values were measured by NIH Image J.

### Serum AP Complement Activity Assays

LPS-stimulated AP activity was performed as previously published by ELISA (46). Briefly, LPS (2 μg/well) was coated on plates. Ten-fold diluted serum with AP buffer was incubated on plates at 37°C for 1 h. Serum diluted with EDTA buffer (2.5 mM barbital, 1.5 mM sodium barbital, 144 mM NaCl, and 50 mM EDTA, pH 7.2–7.4) was used as a negative control. After washing, the plates were incubated with rat anti-mouse C3 antibody and followed by incubation with HRP-conjugated anti-rat IgG. Tetramethylbenzidine (TMB, Solarbio, Beijing, China) was used as substrate and 1 M H_2_SO_4_ was used as a stop solution. The absorbance was measured with Multiskan GO microplate spectrophotometer (Thermo Scientific, Massachusetts, USA).

For the detection of LPS-induced C5a production, 10-fold diluted serum was incubated with 0.125 mg/ml LPS or equal volume of PBS as control at 37°C for 2 h and the reactions were stopped by 50 mM EDTA. The reaction mixtures were added to the plate, which was coated with purified rat anti-mouse C5a antibody (1 μg/ml) and incubated at 37°C for 2 h. The mixtures were discarded and biotin rat anti-mouse C5a (0.5 μg/ml) was added into the according plates as detection antibody. After washing, streptavidin HRP (1:1,000) (BD Pharmingen, California, USA) was added and incubated at 37°C for 1 h. TMB (Solarbio, Beijing, China) was used as substrate and 1 M H_2_SO_4_ was used as a stop solution. The absorbance was measured with Multiskan GO microplate spectrophotometer (Thermo Scientific, Massachusetts, USA).

### LPS *in vivo* Challenge

Six- to eight-week-old male mice were injected intraperitoneally (i.p.) with 10 mg/kg LPS dissolved in PBS and the control group mice were injected i.p. with equal volume of PBS. The EDTA anticoagulant blood was extracted from the tail tip at different time points and the plasma was collected to analyze with Western blotting. Mice were sacrificed at different time points and the EDTA anticoagulant blood was harvested from heart puncture. Partial whole blood was used for complete blood counting using TEK-II Mini full-automatic animal blood analyzer (Tecom Science, Jiangxi, China). The plasma was collected for C3a, C5a, IL-1β and TNF-α concentration detection using ELISA kit as described below.

### Plasma C3a, C5a, IL-1β, and TNF-α Measurements

LPS challenged mice were anesthetized and EDTA anticoagulant blood was collected by heart puncture. The whole blood was centrifuged at 4,000 rpm 4°C for 5 min and the supernatant was collected. Plasma levels of C3a and C5a were determined using ELISA kits (Mybiosource, California, USA) according to the manufacturer's instructions. The dilution factor of C3a and C5a was 1:4. Plasma IL-1β and TNF-α were measured with ELISA kits (R&D Systems, Minnesota, USA) according to the manufacturer's instructions.

### Survival Curve

About 8-week-old mice were injected i.p. with 10 mg/kg LPS. Sixteen hours later, these mice were observed every half hour and the death of mice was recorded. The data were analyzed by GraphPad Prism version 5.0 using the Log-rank (Mantel–Cox) test.

### Histological Analyses

Mice were sacrificed at 12 h after LPS challenge and the left kidneys were harvested. Half of the kidney was fixed in 4% paraformaldehyde for 24 h at 4°C, dehydrated using graded ethanol, cleared with xylene, and embedded in paraffin. Paraffin blocks were sliced into 3-μm sections. Hematoxylin and eosin (HE) staining was performed on the sections using hematoxylin and eosin staining kit (Solarbio, Beijing, China). Pictures were captured with Nicon Ci-L microscope (Nicon, Tokyo, Japan) at 200× magnification. Tubular damage was assessed by tubular dilation, necrosis, and apoptosis. Tubular dilations were counted in every non-overlap field and averaged. Cell necrosis and apoptosis were determined using *in situ* cell death detection kit (Roche, Basel, Switzerland). The fluorescent pictures were captured with an LSM 710 confocal fluorescence microscope (Zeiss, Oberkochen, Germany) at 100× magnification. Only dots overlapped with nuclei were counted and the whole cell number of each field was counted by Image J. Proportion of apoptotic and necrotic cells was computed.

### Immunofluorescent Staining of Fibrin

Paraffin slides that were processed by xylene de-waxing and gradient ethanol hydrating were blocked by ready-to-use goat serum (Boster, Wuhan, China) at 37°C for 1 h and incubated with rabbit anti-fibrinogen (1:400) overnight at 4°C. After washing three times with PBS, the slides were incubated with Alexa Fluor 488 goat anti-rabbit IgG (1:1,000) (Invitrogen, California, USA) at 37°C for 1 h. The slides were washed three times and mounted with mounting medium with DAPI (ZSGB-Bio, Beijing, China). Images were captured using Leica TCS SP5 confocal laser scanning microscope (Leica Microsystems, Wetzlar, Germany) at 200× magnification. The fluorescence intensity and fluorescence positive area were analyzed by Image J.

### Cluster Analysis and Homology Analysis

The protein sequences were downloaded from NCBI-Protein (https://www.ncbi.nlm.nih.gov/protein/). Amino acid sequences were aligned by ClustalW with MEGA 7.0 (47). The alignment diagram was drawn with GeneDoc. The evolutionary tree was constructed using the Neighbor-Joining method with Mega 7.0 (47, 48). The domain structures of FHRs were analyzed by SMART (http://smart.embl-heidelberg.de/). Domain similarities among different FHRs were computed with pBLAST.

### Statistics

Data were presented as mean ± SEM. Student's *t*-test was used to compare the two groups. Time course of factors of different time points after LPS challenge was analyzed with one-way ANOVA by SPSS software. Comparison of survival curves was analyzed by Mantel–Cox test. Significant differences were considered when the *p*-value was less than 0.05 and extremely significant differences were considered when the *p*-value was less than 0.01.

## Results

### Homology Analysis of Human and Mouse FHR Proteins and Generation of *Cfhr1* Knockout (KO) Mice

To ascertain the mouse FHR-E homolog of human FHR proteins, homology analysis between mouse and human FHRs was conducted. The evolutionary tree of FHRs showed that FHR-E converged with human FHR-1, FHR-2, and FHR-5 (Figure 1A). The overall homology between mouse FHR-E and human FHR-1/2/5 is about 70%, while FHRB and FHRC have about 40% homology to FHR-1/2/5 (40). Structure analyses of FHR-E and human FHR-1, FHR-2, and FHR-5 by SMART showed that they all consist of CCP modules. Amino acid sequence of each CCP in FHR-E was compared with human FHR-1, FHR-2, and FHR-5 by pBLAST and amino acid sequence alignment was analyzed by MEGA 7.0. Results showed that FHR-E shared more similarity with human FHR-1 (Figures 1B–E). With this regard, we selected the mouse *Cfhr1* gene that encodes the FHR-E protein for the generation of a gene knockout colony.

The exons from 1 to 5 of *Cfhr1* were deleted by using the CRISPR/Cas9 system on C57BL/6 mouse (Figure 2A). Verification of *Cfhr1* deletion was conducted at DNA, mRNA, and protein levels (Figures 2B–D). The results demonstrated that FHR-E was detectable in murine plasma and was depleted in the knockout mice. Our homemade anti-FHR-E antibody recognized murine FH as well due to the high epitope identities to SCRs 19 and 20 of FH (data not shown). This is the first report of FHR-E in murine plasma. FHR-B and FHR-C has been detected in mouse plasma by using FH-specific antiserum (38). The unrecognized FHR-E in their experiment may be because of the antibody specificity. Both heterozygotes (*Cfhr1*^−^^/+^) and homozygotes (*Cfhr1*^−^^/−^) were viable and healthy bred in SPF (specific pathogen free) mouse facility.

### FHR-E Deficiency Did Not Affect the Physiological Intrinsic AP Regulation

**Figure 3:**
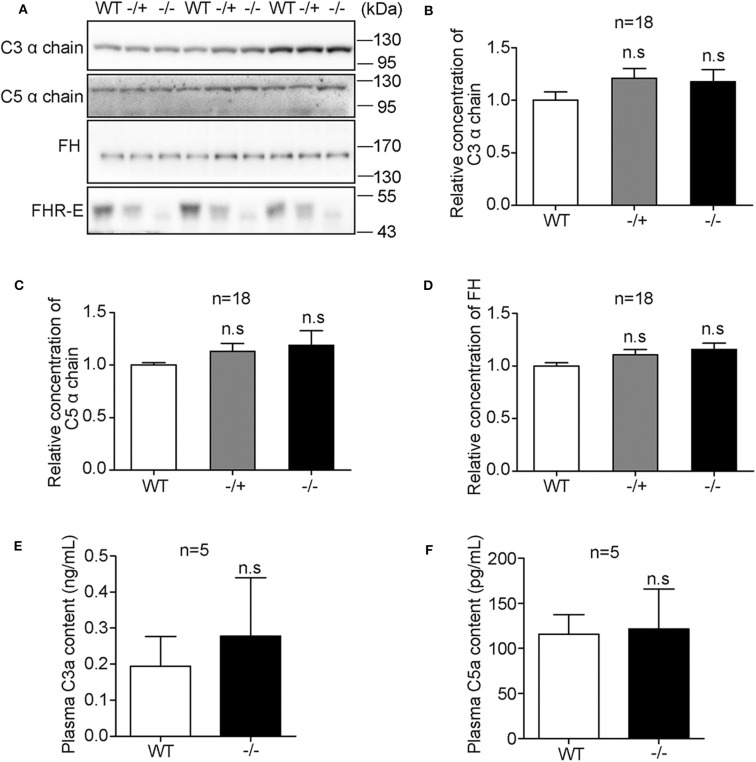
Detection of plasma levels of major complement factors among mice of different genotypes under physiological conditions. **(A)** Representative Western blotting analyses of plasma C3, C5, FH, and FHR-E. Equal volume of plasma was loaded. Eighteen mice in each group were included. **(B–D)** The gray values of C3, C5, and FH were measured with Image J. The calculated gray values of each blot were divided by the average gray value of wild type in the blot. **(E,F)** Measurements of plasma concentration of C3a and C5a with ELISA kit. EDTA anti-coagulant blood was obtained from heart puncture. Five mice in each group were used. Very low background absorption was detected in different genotype mice. n.s., not significant.

Under physiological condition, AP keeps at a low level of activation by spontaneous hydrolysis of C3 (49). To study whether *Cfhr1* deletion can trigger complement dysregulation under physiological condition, the plasma levels of C3, C5, and FH, central molecules of complement and main soluble regulator of AP, were detected on 6- to 8-week-old male sibling mice. Western blotting results showed no obvious changes among mice of different genotypes (Figure 3A). The relative gray values of 18 mice were analyzed and results showed that there were no significant differences (Figures 3B–D). Furthermore, the concentrations of C3a and C5a, which are small cleaved fragments generated by complement activation, and the key mediators of inflammation were measured. Neither the level of C3a nor C5a had significant differences among different genotypes (Figures 3E,F). These results suggested that under physiological condition, the deletion of *Cfhr1* was not sufficient to impact the AP regulation.

### AP Activation Is Enhanced in *Cfhr1^−/−^* Mice in Response to LPS Treatment *in vitro*

**Figure 4:**
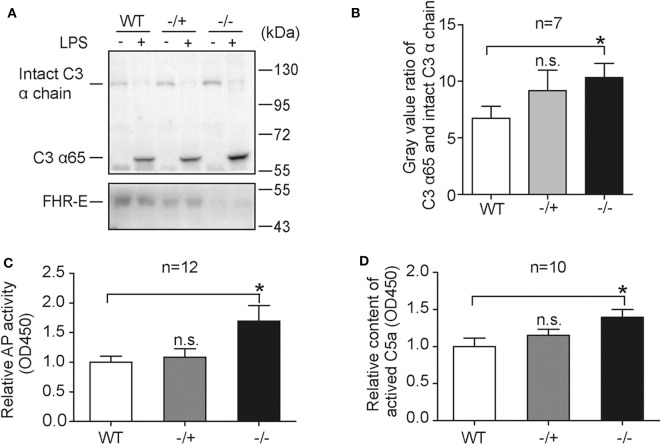
Serological measurements of *in vitro* LPS stimulation. **(A)** Representative Western blotting analysis of C3 activation by LPS treatment among mice of different genotypes. Western blotting analysis of FHR-E was used for genotyping. Equal volumes of PBS were used as negative controls. Equal volume of protein was loaded. Seven mice in each group were used. **(B)** The gray values of C3 α65 fragment and intact C3 α chain in **(A)** were measured with Image J. The gray value of C3 α65 was divided by the gray value of intact C3 α chain in the same lane. The ratio of C3 α65 and intact C3 α chain was used to represent cleavage status of C3 α chain. **(C)** Detection of AP activation by plate bounded LPS in diluted serum with ELISA method. Equivalent LPS was coated on the plate. AP buffer and EDTA buffer diluted serum was added into the plate and incubated for 1 h at 37°C. EDTA buffer diluted serum was used as a negative control for background subtraction. After washing, the LPS-bound C3b was detected with C3 antibody. Twelve mice in each group were used. **(D)** Measurement of the concentration of C5a produced at 2 h after LPS *in vitro* stimulation. AP buffer diluted serum was incubated with LPS or PBS for 2 h at 37°C. Equal volume of PBS was used as a negative control for background subtraction. The quantity of C5a was detected with sandwich enzyme-linked immunosorbent assay. Ten pairs of mice were used. n.s., not significant. *p < 0.05.

LPS, the principal component of gram-negative bacteria cell wall, is an activator of AP (50) and responsible for gram-negative bacteria induced sepsis (51). To test the effect of FHR-E deficiency on LPS-induced AP, *in vitro* LPS-induced AP activation experiments were performed. LPS was incubated with serum from mice of different genotypes and the mixtures were analyzed by Western blotting. A higher amount of C3-activated fragment in *Cfhr1*^−^^/−^ mice was observed compared to the wild-type group (Figures 4A,B). AP activity measured by ELISA assay (46, 52) was also conducted to verify this effect. As expected, *Cfhr1*^−/−^ mice showed more deposited C3b on LPS (Figure 4C). Furthermore, LPS-induced C5a production, an ensuing event of AP activation, was determined and significantly higher concentration of C5a was detected in *Cfhr1*^−/−^ mice compared to wild-type mice (Figure 4D). An increasing tendency was observed in the heterozygotes, but there was no significant difference between WT and heterozygotes, suggesting that dose effects of FHR-E in heterozygotes may exist. Thus, FHR-E deficiency promoted LPS-induced AP activation *in vitro*.

### FHR-E Deficiency Resulted in Enhanced Activation of Complement Pathway and Inflammation *in vivo*

**Figure 5:**
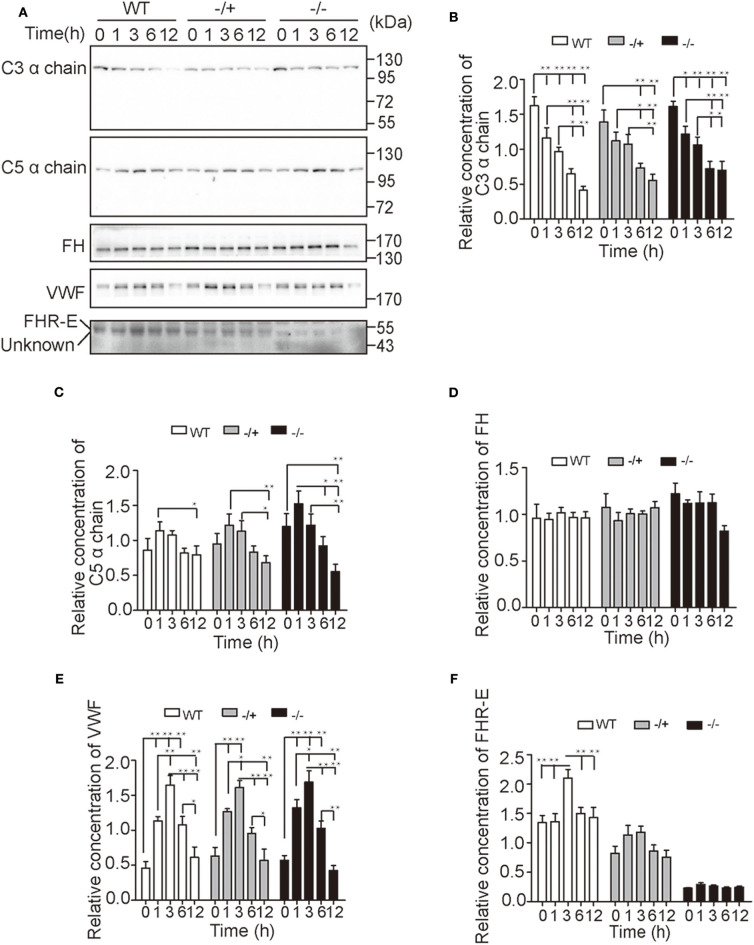
Detection of major complement and coagulation factors after *in vivo* LPS challenge. **(A)** Time-course changes of C3, C5, FH, VWF, and FHR-E expression levels analyzed by Western blotting. Equal volume of plasma was loaded. Five mice in each group were used. “Unknown” means unknown proteins recognized by the anti-FHR-E antibody. **(B–F)** The gray value quantification of C3, C5, FH, VWF, and FHR-E in **(A)** measured with Image J. The gray values of each membrane were divided by the average gray value of all samples on the membrane. The data were analyzed with one-way ANOVA. *p < 0.05, **p < 0.01.

To further study the regulatory function of FHR-E, *in vivo* challenge of LPS was applied. EDTA anticoagulant blood of different time points was collected from the tail tip to monitor the content changes of main complement or coagulation factors using Western blotting. Our results showed that the level of C3 decreased over time and that the level of C5 increased at first and decreased from 1 h after LPS challenge. No significant difference was observed among different genotypes at different points. However, more significant decrease of C5 content over time was observed in *Cfhr1*^−/−^ mice compared with wild-type mice (Figures 5A–C). The levels of FH and von Willebrand factor (VWF), which is involved in hemostasis, were also determined. The content of VWF increased at first and decreased from 3 h post LPS challenge (Figures 5A,E) while the content of FH did not have any observable change over time (Figures 5A,D). This demonstrated that the hemostasis was promptly triggered and gradually vanished as the exhaustion of clotting factor, and that FHR-E deficiency and LPS stimulation did not affect the level of FH. The contents of FHR-E in wild-type and heterozygous mice were also measured. Interestingly, we found that FHR-E increased significantly at 3 h post-LPS challenge (Figures 5A,F). The data in Figure 5 were obtained from the same batches of mice.

**Figure 6:**
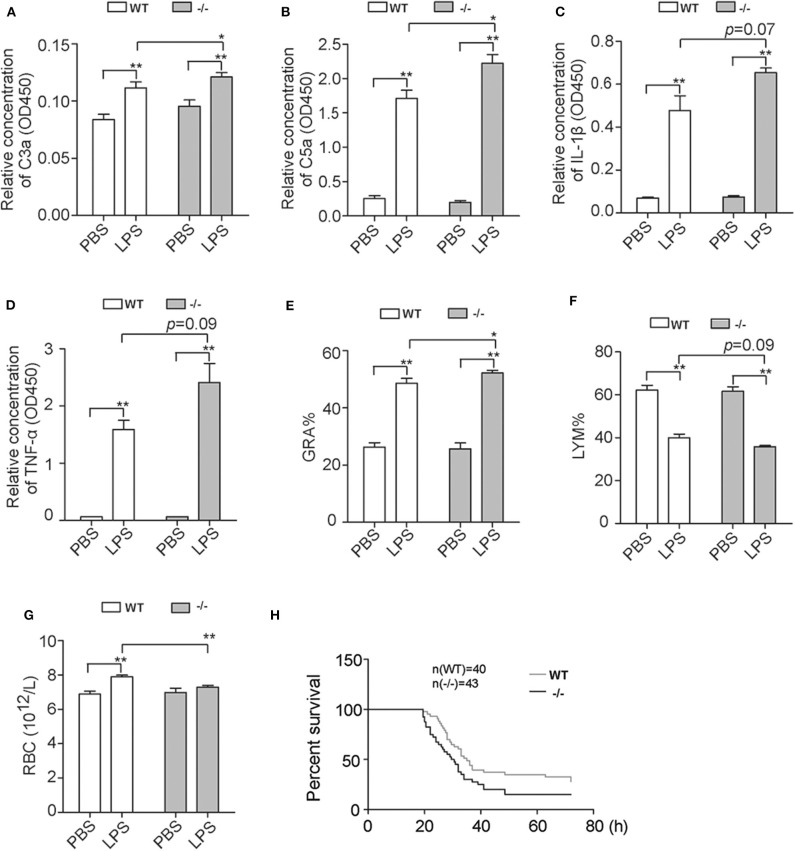
Detection of complement-activated products and blood cells after *in vivo* LPS challenge. **(A,B)** C3a and C5a concentration measurements with ELISA kit at 3 h after *in vivo* LPS challenge. Four mice in each group were included. **(C,D)** IL-1β and TNF-α concentration measurements with ELISA kit at 3 h after *in vivo* LPS challenge. Three mice in each group were included. **(E–G)** Percentages of granulocytes and lymphocytes at 6 h after LPS challenge and red cells at 12 h after LPS challenge measured by animal blood analyzer. GRA, granulocytes. LYM, lymphocytes. RBC, red blood cells. Four mice in each group were included. **(H)** The survival data of two groups with i.p. injection of 10 mg/kg LPS. The data were analyzed by Gehan–Breslow–Wilcoxon test. The number of wild-type mice and *Cfhr1*^−/−^ mice was 40 and 43, respectively. *p < 0.05, **p < 0.01.

Furthermore, another group of mice were challenged with LPS and EDTA anticoagulant blood of different time points was harvested from heart puncture. The contents of plasma C3a and C5a of *Cfhr1*^−/−^ mice were significantly higher than wild-type controls at 3 h post-challenge (Figures 6A,B). These results demonstrated that FHR-E deficiency accelerated LPS-induced AP. The plasma IL-1β and TNF-α contents at 3 h post-challenge were detected and elevated concentrations of IL-1β and TNF-α were found in *Cfhr1*^−/−^ mice (Figures 6C,D). Subsequently, we observed that the proportion of granulocytes significantly increased and lymphocytes mildly decreased at 6 h after challenge, which suggests that *Cfhr1*^−/−^ mice had more severe inflammation (Figures 6E,F). The quantity of red blood cells in *Cfhr1*^−/−^ mice at 12 h after LPS challenge was dramatically lower than the wild-type mice, which indicates that much more congestion could happen in *Cfhr1* deletion mice (Figure 6G). In summary, the *Cfhr1*^−/−^ mice had features of enhanced complement activation and inflammation, which may lead to organ injury.

To explore the endpoint effect of FHR-E deficiency on LPS-induced sepsis, the mortality assay was performed. *Cfhr1*^−/−^ and wild-type mice were administrated i.p. injection of 10 mg/kg LPS. The survival data were recorded and analyzed. Compared to the wild-type mice, the average survival time of *Cfhr1*^−/−^ mice was significantly shorter and the mortality rate of *Cfhr1*^−/−^ mice was significantly higher (Figure 6H; *p* = 0.0425, *n* = 43 in the wild-type group, *n* = 40 in the *Cfhr1*^−/−^ mice group). Median survival time of wild-type and *Cfhr1*^−/−^ mice was 35 and 30 h, respectively. At 26 h post-LPS challenge, 14% of wild-type mice died while 35% of *Cfhr1*^−/−^ mice died. Thus, FHR-E plays a protective role on LPS-induced sepsis.

### FHR-E Deficiency Promoted LPS-Induced AKI

**Figure 7:**
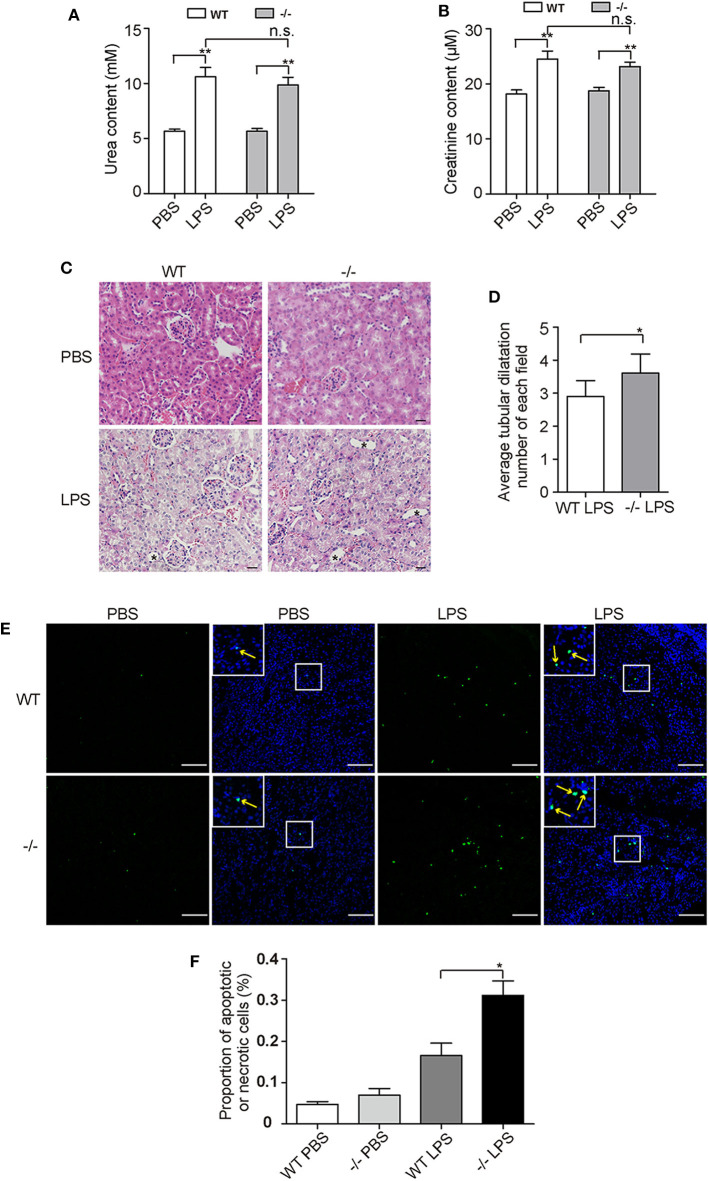
Tests of kidney injury at 12 h after LPS challenge. Three mice in each group were used. **(A,B)** Measurements of urea and creatinine content. **(C)** Hematoxylin and eosin (HE) staining of kidney sections. Pictures were taken at a magnification of 200×. The slice thickness was 3 μM. The bar is 20 μM. Tubular dilations were marked by asterisks. **(D)** Average tubular dilation number of each field. Non-overlapped fields of one section were counted and averaged. **(E)** TUNEL staining of kidney sections. Pictures were taken at a magnification of 100×. The green signals indicate nuclei of apoptotic or necrotic cell whose DNA strand breaks in nuclei were labeled by fluorescein-dUTP. The blue signals indicate total nuclei, which were stained with DAPI. The field in the upper left corner is the zoom picture in the white square box. Arrows point to the fluorescence positive points. The bar is 100 μM. **(F)** Proportion of apoptotic and necrotic cells. Green fluorescence positive points that were overlapped with blue points of 10 non-overlapped fields were counted. Total nuclei numbers were recognized as total cell numbers counted by Image J. The ratio of fluorescence nuclei among total nuclei was calculated. *p < 0.05, **p < 0.01, n.s., not significant.

**Figure 8:**
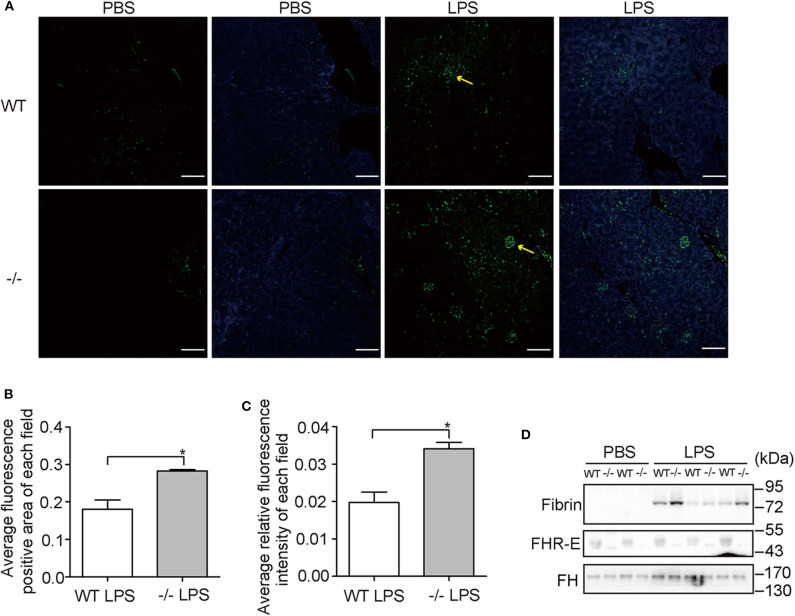
Vascular damage examinations. Three mice in each group were used. **(A)** Immunostaining of fibrin on kidney sections. Pictures were taken at a magnification of 200×. The green signals indicate fibrin. The blue signals indicate total nuclei stained with DAPI. Arrows point to the fluorescence positive area. The bar is 50 μM. **(B)** Comparison of fluorescence positive area between WT and *Cfhr1*^−/−^ mice. Fluorescence positive area of each field was calculated by Image J. Twenty non-overlapped fields were counted and averaged. **(C)** Comparison of fluorescence intensity between wild-type and *Cfhr1*^−/−^ mice. Fluorescence intensity of each field was calculated by Image J. Twenty non-overlapped fields were counted and averaged. **(D)** Western blotting analysis of plasma fibrin of 12 h after LPS challenge. Western blotting of FHR-E was performed to determine the genotypes. Equal volumes of plasma were loaded. The Western blotting of FH on the same blot was regarded as an internal control. *p < 0.05.

Human *CFHR1* deletion was associated with nephropathy (27, 53) and kidney injury can be induced by complement overactivation (54). Urea and creatinine, which are indicators of renal function, were tested at 12 h post-challenge and significant increases were observed in both WT and KO groups (Figures 7A,B). However, we did not observe an apparent increase in the KO group compared with the WT group after LPS treatment. This suggests that renal function was not worsened dramatically in FHR-E deficiency at the time of measurement. We then performed histological examination of kidney. Renal tissue sections of 12 h after LPS challenge were prepared and HE staining was conducted. Kidney injury degree was assessed by the tubular dilations, apoptosis, and necrosis. Tubular dilations were quantified, and cell apoptosis and necrosis were determined through TUNEL assay. More tubular dilations (Figures 7C,D) and more cell apoptosis and necrosis (Figures 7E,F) were exhibited in *Cfhr1*^−/−^ mice. Fibrin, which is the marker of vascular injury, was also stained to testify tissue injury. We found that *Cfhr1*^−/−^ mice had significantly more fibrin deposition than wild-type mice (Figures 8A–C). Plasma fibrin was also analyzed by Western blotting and higher concentration of plasma fibrin was found in *Cfhr1*^−/−^ mice (Figure 8D).

## Discussion

In this study, *Cfhr1* knockout mice were generated to further investigate its role in AP regulation. No obvious changes of C3a and C5a concentration in plasma were found in *Cfhr1* knockout mice. These results suggest that the basal level of FHR-E plays negligible roles in spontaneously activated AP pathway. This may explain why many individuals with *CFHR1* homozygous deletion are healthy (34). Surprisingly, with FHR-E deficiency, increased C3 and C5 cleavage were found after LPS stimulation *in vitro*, suggesting that FHR-E itself inhibits LPS-induced AP activation. This may be explained by the conformation switch model in which soluble FH exists in a low-affinity latent conformation and transits to high-affinity activated conformation by interacting with self-surface targets (55). Based on this model, FH may play negligible inhibition on LPS-induced AP and FHR-E may compete C3b with positive regulators of AP, like properdin, which plays an essential role in LPS induced AP activation (46). When FHR-E is absent, AP activity is enhanced by the positive regulators. The inhibitory effect of FHR-1 on C5 convertase has been reported (30) and was regarded as a therapeutic target (56). The result that more C5a production after LPS challenge in *Cfhr1*^−/−^ mice was observed supports the idea that FHR-E has functional homology to human FHR-1.

*CFHR1* deletion has been reported to be associated with renal disease in an incomplete penetrance and a second hit such as infection is considered to be essential for disease development (57). It is unknown whether *CFHR1* homozygous deletion alone would trigger the onset of these conditions upon infection. One reported case showed that shiga toxin was a potential trigger of *CFHR1* deletion-related thrombotic microangiopathy (58). Here, we chose LPS, which is the main constituent of the wall of gram-negative bacteria as a stimulator to study the effect of *Cfhr1* deletion in mice for possible pathological changes. Both *in vitro* and *in vivo* assays showed that FHR-E deficiency significantly promoted LPS-induced AP activation. These results suggested that FHR-E may play a coordinated role with FH in determining complement activation. However, the mechanism of selective activation of AP remains enigmatic. In many aHUS patients with *CFHR1* deletion, FH autoantibody was detectable (27). It was believed that aHUS may result from the blocking of FH function by FH auto-antibody. However, this cannot explain those aHUS patients with *CFHR1* deletion without FH auto-antibody (59). Our results suggest that FHR-E deficiency promotes infection-induced damage through enhancing AP activation. This may provide a hint for FHR-1 deficiency-related nephropathy. Nevertheless, how FHR-E functions in this complex process warrants further investigation. In addition, we did not see much difference of FH level in FHR-E deficiency mice.

To the best of our knowledge, there has been no report of FHRs on any inflammatory disease mouse models. Our results revealed that *Cfhr1* knockout mice challenged with LPS served as a good model to study the intricate network of complement, inflammation, and coagulation in sepsis and AKI. We found that *Cfhr1*^−/−^ mice showed higher content of C3a and C5a, more severe inflammation, more blood coagulation, and more severe renal injury after LPS challenge compared with the wild-type group. However, we did not see significantly higher levels of urea and creatinine in *Cfhr1*^−/−^ mice compared to the wild-type mice upon LPS challenge. C3a and C5a, which are two main products of complement activation, have been shown to have anti-inflammatory (60, 61) and pro-inflammatory function (62), respectively. Administration of C5a receptor antagonist peptide in thrombotic glomerulonephritis model significantly reduced leucocyte accumulation and thrombus formation in glomeruli (63) and improved survival of mice with sepsis (64), while administration of C3a receptor antagonist exacerbated mortality of mice with sepsis (64). The regulation mechanism of C3a and C5a on sepsis development, which seemed to have converse effect, is still unclear. In our study, the deficiency of FHR-E promoted complement activation with more C3a and C5a production, which in turn induced more inflammation and eventually accelerated coagulation and tissue injury. These results suggested that C5a may play a more potent role on sepsis development. Our *Cfhr1*^−/−^ mouse model provides a unique tool to study the underlying mechanism in which AP is activated upon LPS challenge to induce tissue injury. Beyond the scope of this study, it is speculative that patients with FHR deficiency may be susceptible to severe conditions under SARS-CoV-2 infection when sepsis is induced in COVID-19 patients.

## Data Availability Statement

The datasets generated for this study are available on request to the corresponding author.

## Ethics Statement

The animal study was reviewed and approved by Institutional Animal Care and Use Committee of IGDB (Institute of Genetics and Developmental Biology, Chinese Academy of Science).

## Author Contributions

XLi, ZH, and WL designed the experiments, analyzed the data, and supervised the project. XLiu contributed to the initiation and design of the project. XLi and WL wrote the manuscript. XLi and ZH performed the assays.

## Conflict of Interest

The authors declare that the research was conducted in the absence of any commercial or financial relationships that could be construed as a potential conflict of interest.
